# Can Hyperspectral Imaging and Neural Network Classification Be Used for Ore Grade Discrimination at the Point of Excavation?

**DOI:** 10.3390/s22072687

**Published:** 2022-03-31

**Authors:** Krystian A. Choros, Andrew T. Job, Michael L. Edgar, Kevin J. Austin, Peter Ross McAree

**Affiliations:** 1 School of Mechanical and Mining Engineering, The University of Queensland, Brisbane, QLD 4072, Australia; andrew.job@plotlogic.com (A.T.J.); kevin.austin@uq.edu.au (K.J.A.); p.mcaree@uq.edu.au (P.R.M.); 2 Plotlogic Pty Ltd., 12 Thompson St., Bowen Hills, QLD 4006, Australia; mick.edgar@plotlogic.com

**Keywords:** ground-based hyperspectral imaging, machine learning, deep learning, ore characterization, gold deposits

## Abstract

This work determines whether hyperspectral imaging is suitable for discriminating ore from waste at the point of excavation. A prototype scanning system was developed for this study. This system combined hyperspectral cameras and a three-dimensional LiDAR, mounted on a pan-tilt head, and a positioning system which determined the spatial location of the resultant hyperspectral data cube. This system was used to obtain scans both in the laboratory and at a gold mine in Western Australia. Samples from this mine site were assayed to determine their gold concentration and were scanned using the hyperspectral apparatus in the laboratory to create a library of labelled reference spectra. This library was used as (i) the reference set for spectral angle mapper classification and (ii) a training set for a convolutional neural network classifier. Both classification approaches were found to classify ore and waste on the scanned face with good accuracy when compared to the mine geological model. Greater resolution on the classification of ore grade quality was compromised by the quality and quantity of training data. The work provides evidence that an excavator-mounted hyperspectral system could be used to guide a human or autonomous excavator operator to selectively dig ore and minimise dilution.

## 1. Introduction

Metal mining operations benefit from precise knowledge of ore grade at the point of excavation to minimise waste dilution and maximize ore recovery. Presently, mine models are constructed from drill core sampling and analysis, which requires significant expertise and is available only at discrete sample points. Precise information about ore grade between drill locations or post-blast disturbance is not available until after the ore has been processed. If grade information were known in advance, it would allow for more efficient ore recovery. This paper explores the application of hyperspectral imaging to provide precise information at the time of excavation.

Hyperspectral imaging is the acquisition of contiguous spectral information for each pixel in a 2D image [1]. Early work by Hunt [2] described the electronic and vibrational processes that create absorption features at certain wavelengths in the reflectance or emittance spectra of minerals. This information acts as a spectral signature; minerals at the surface of the scanned objects can be identified by the position, depth, width, and asymmetry of spectral absorption features [3]. Many methods have been developed for the characterisation of minerals from their spectral signatures, summarised by Asadzadeh and de Souza Filho [4]. They describe two broad categories of spectral processing methods: (i) knowledge-based, i.e., using knowledge of the characteristic absorption features to identify minerals directly from their spectra; or (ii) data-based, i.e., using reference data to classify target data, such as by direct comparison of spectral similarity to a reference library of labelled spectra. This is a useful distinction of the approaches to spectral analysis; the development of robust data-based methods reduces the requirement for specialised knowledge of the spectral absorption features relevant to the mineral characterisation of an individual mine site.

Much of the interest in hyperspectral imaging for geological applications has been in the field of remote sensing, as summarised by van der Meer et al. [5] and, more recently, Peyghambari and Zhang [6]. Dedicated hyperspectral sensing platforms and facilities such as airborne sensors (e.g., AVIRIS [7]) and spaceborne sensors (e.g., Hyperion [8] and EnMAP [9]) provide a wealth of data for researchers. However, greater availability of hyperspectral sensors has led to ground-based applications, which enables the use of hyperspectral imaging within mining operations. Kurz et al. [10] demonstrated the use of a terrestrial short-wavelength infrared (SWIR) sensor and LiDAR to classify and identify minerals in aboveground and subsurface applications. This is an early example of LiDAR and hyperspectral data fusion applied to face mapping. Okyay et al. [11] demonstrated end-member-based classification on a ground-based scan of a road cut, using both laboratory spectra (from samples obtained in the field during scanning) and spectra in the field image.

A number of authors have considered fusion of hyperspectral and LiDAR data to address various problems that include registering hyperspectral data to LiDAR data to improve geological interpretation and accuracy [12,13,14,15,16,17,18,19], illumination correction [20,21], assessing slope stability [22], and to localise data in airborne applications [23,24]. In this paper, LiDAR in combination with GNSS is used to spatially locate hyperspectral pixels within the surveyed scene.

The use of two hyperspectral cameras to cover both visible-near-infrared (VNIR) and SWIR spectra brings the challenge of image fusion in panoramic push-broom operation. Okyay and Khan [25] co-registered images from VNIR and SWIR cameras using the scale-invariant feature transform algorithm, also developing a correction for VNIR spectral information that removes discontinuities between the spectra of each camera. Krupnik and Khan [26], in addition to a review of terrestrial applications, presented three case studies of VNIR and SWIR imaging in mine environments, with spectral feature mapping used to identify minerals. Barton et al. [27] presented a case study of field mapping and classification using spectral angle mapper (SAM) classification with both ground-based and unmanned aerial vehicle (UAV) sensing. In particular, they explored the potential to integrate hyperspectral imaging into existing mining operation and interface with common geological software.

Many of these field applications have focused on mineral identification. Additional benefit can be achieved by relating spectral features to ore grade. Dalm et al. [28] used principal component analysis to discriminate sub-economic-grade copper in SWIR images of samples from a copper mine. Dalm et al. [29] analysed near-infrared and SWIR drill core samples of an epithermal gold–silver deposit and attempted to segment the set into ore and waste, succeeding in segmenting a population of waste samples. Both of these studies successfully identified a subset of the waste or sub-economic grade samples, but failed to determine a metric that correctly classified both ore and waste. Lypaczewski et al. [30] analysed core samples from a gold deposit and identified spectral features correlating with gold content. These spectral analysis methods are knowledge-based methods, requiring expertise in spectral feature analysis and a good understanding of the regional geology.

Recent developments in machine learning for hyperspectral imaging classification (summarised in [31]) present an opportunity to apply data-based techniques for ore/waste discrimination, without the manual work of identifying spectral features. A promising candidate method is based on convolutional neural networks (CNNs), and there is a history of using CNNs to classify hyperspectral data in remote sensing applications [32,33,34,35,36,37,38,39]. This approach is generally recognised to perform well, but there have been few applications in ground-based hyperspectral sensing or geological applications. Notably, Windrim et al. [40] applied transfer learning to pretrain a CNN on large labelled datasets, then fine-tuned the classifier on a domain-specific dataset, demonstrating classification of a ground-based hyperspectral scan obtained at a mine site. This acknowledges and addresses some of the difficulties of obtaining adequate well-labelled datasets and ground truth information for supervised classification problems.

Job et al. [41] presented a desktop study of a shovel-mounted hyperspectral imaging system for coal or ore sensing. The intent was to leverage the motion of the machine to conduct a spectral survey of the working face during operation. Herein, we take the first steps towards proving this capability in a field deployment within a production gold mine. We evaluate the application of a convolution neural network, trained on samples obtained from the mine, to discriminate ore and waste at the point of excavation. The performance of the neural network is compared with results from the spectral angle mapper as a competing approach and with the mine site geological model. The results suggest that ore grade discrimination is possible and an instrument mounted on the excavator could provide real-time information on ore–waste boundaries at the point of excavation. This information could be provided to the excavator operator, or the planning algorithms of an automation system, to reduce dilution and increase ore recovery.

## 2. Materials and Methods

### 2.1. Hardware

**Figure 1:**
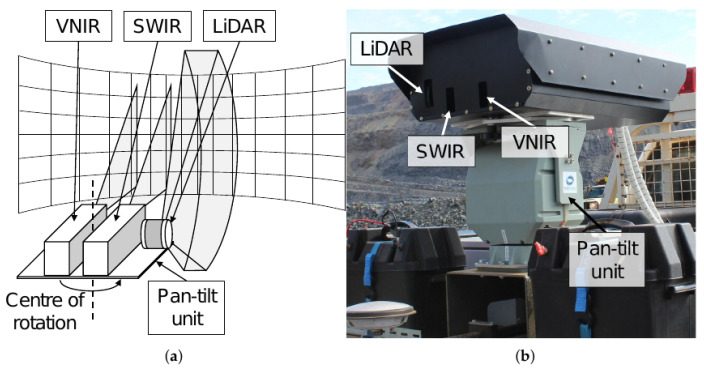
(**a**) Schematic of sensing apparatus showing visible-near-infrared (VNIR) and short-wavelength infrared (SWIR) hyperspectral cameras, LiDAR, and pan-tilt unit. (**b**) Sensing apparatus mounted on a utility vehicle. The hyperspectral cameras and LiDAR are protected by a sun-shading enclosure, shown sitting atop the pan-tilt unit.

The apparatus to collect data for the study comprised VNIR and SWIR hyperspectral cameras and a three-dimensional light detection and ranging (LiDAR) sensor mounted on a pan-tilt head. Real-time kinematic global navigation satellite system (RTK GNSS) receivers and joint encoders from the pan-tilt head provided information to determine the six-degree-of-freedom (DOF) pose of the camera–LiDAR assembly. The apparatus was mounted to a utility vehicle, allowing transportation around the mine site and a platform from which to perform surveys (see Figure 1).

The survey process consisted of three steps: (1) a LiDAR survey of the working face to establish a 3D terrain model; (2) projection of spectral information onto the terrain model as the cameras were swept across the scene; and (3) geolocating the survey relative to the mine map using the platform pose provided by RTK GNSS receivers. The result of this process is a hyperspectral cube with two spatial dimensions and a third spectral dimension. In this study, the spatial dimensions are defined by spherical coordinates, which provide a convenient mapping with the movement of the sensors.

Terrain data were acquired with a Velodyne VLP-16 [42], a 16-beam rotating LiDAR, that provided 360${}^{\circ }$ range in the rotating axis and 30${}^{\circ }$ field of view (FOV) in the azimuth axis. The LiDAR was mounted sideways on the pan-tilt platform so the beams rotated in the vertical plane. Three RTK GNSS receivers were used to obtain a six-DOF pose solution to orient the system with respect to the mine map, so the final terrain model and hyperspectral data or classified solution were able to be integrated with existing mine models.

Hyperspectral data were acquired with a HySpex VNIR-1800 [43], covering 400–1000 nm with 186 bands (3.26 nm spectral resolution), and a HySpex SWIR-384 [44], covering 950–2500 nm with 288 bands (5.45 nm spectral resolution), from Norsk Elektro Optikk. The VNIR-1800 has a 17${}^{\circ }$ FOV, with a spatial resolution of 0.16 mrad across track and 0.32 mrad along track. The SWIR-364 has a 16${}^{\circ }$ FOV of view, with a spatial resolution of 0.73 mrad both across and along track. Both cameras are line scanners and operate in a push-broom fashion to obtain a complete two-dimensional scan. The cameras were mounted in stereo configuration on the same rotating platform as the LiDAR. Each line scan was an average of 3–5 sequential exposures. Exposure was adjusted so that no pixels in the scene were saturated. The pan speed was adjusted to ensure a contiguous image at the resolution of the cell grid used to store the hyperspectral data.

Two Labsphere Spectralon^®^ targets with 99% and 50% reflectance were placed in the scanned scene to allow the reflectance values to be computed for materials in the scene.

Each line scan was registered to the terrain as it was acquired. Given the known location of the cameras and LiDAR units on the platform, and the known pan and tilt angles of the rotating platform, the position and orientation of the focal point of the cameras were known relative to the origin of the sensing platform frame of reference. Camera calibration information provided the precise field of view of each spatial camera pixel. Hence, each pixel in the line scan was treated as a vector emanating from the focal point of the camera, and was ray cast to find the intersection with the terrain. The hyperspectral data were stored in a spherical cell grid representation which matched the resolution of the SWIR camera. Data from the VNIR camera were downsampled by computing the mean of spectra that were ray cast to the same cell.

### 2.2. Fieldwork

**Figure 2:**
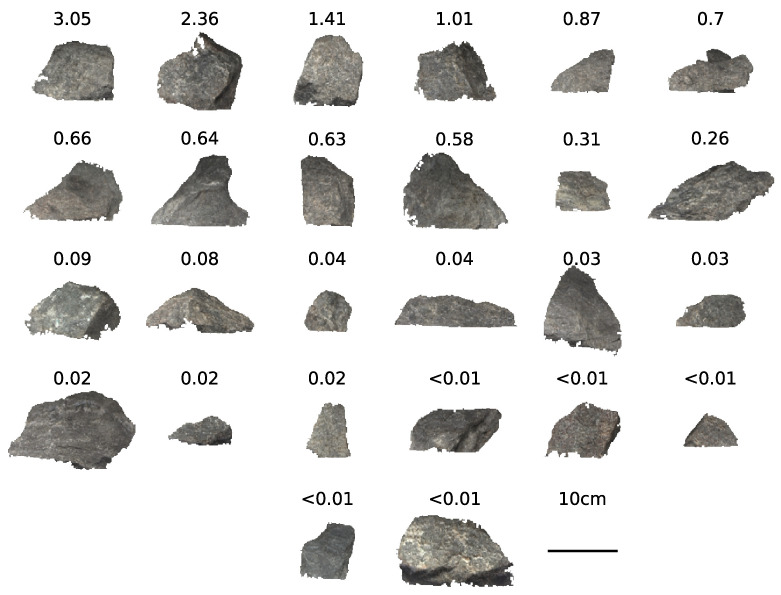
Approximate true colour image of gold ore samples scanned in the laboratory, labelled by assayed gold concentration (ppm). Red, green, and blue channels are the 643 nm, 550 nm, and 464 nm bands from the visible-near-infrared (VNIR) camera.

Fieldwork was undertaken in May 2019 at a gold mine in the Goldfields–Esperance region of Western Australia. Prior to this field work, the mine provided various ore samples, intending to cover a range of ore grades (see Figure 2). These were scanned in a laboratory under broad-spectrum halogen illumination. Scans were obtained at a sample distance of 1 m, using the same system as the field scans except for shorter focal-length lenses. These samples were sent to a laboratory for fire assay to determine gold concentration. The hyperspectral scans of these samples, and the associated concentration data, form the spectral libraries and training sets used for classification, as described in Section 2.4.

**Figure 3:**
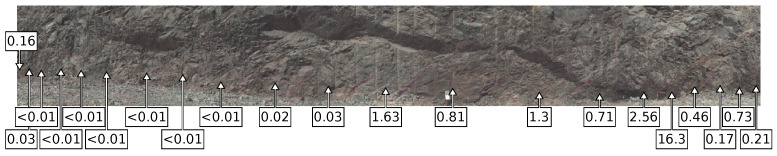
False, three-colour image of the scanned site, with approximate sample locations and gold concentration (ppm) obtained from fire assay. Gold content data provided by AngloGoldAshanti. The red, green, and blue channels display the 1000 nm, 550 nm, and 464 nm bands from the data cube, respectively. The 1000 nm channel from the short-wavelength infrared (SWIR) camera was selected to create an image that fuses information from both cameras.

Figure 3 shows an image of a scanned mine face obtained during fieldwork. The hyperspectral survey was obtained with the equipment positioned 25 m from the face, with a 120${}^{\circ }$ pan angle from start to finish. This scan took 5 min to obtain. The resulting scan has 349 pixels across track and 2577 pixels along track, with a resolution of 0.8 mrad. The maximum distance to the scanned face is 60 m at the extents of the scan, and the height of the face captured by the survey varies from 7 to 13 m across the scan. The spatial resolution of each pixel varies from 2 to 5 cm depending on rotation and distance from the scanned face. The root mean square error of registration between the VNIR and SWIR images, determined from 70 manually selected tie points spanning the scene, is approximately 2/3 of a pixel.

**Figure 4:**
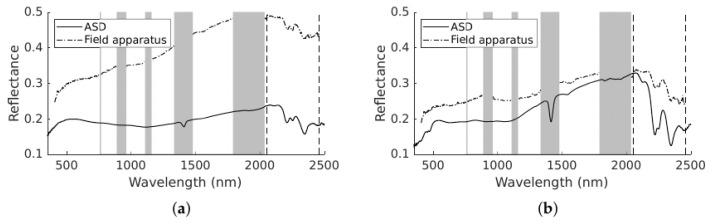

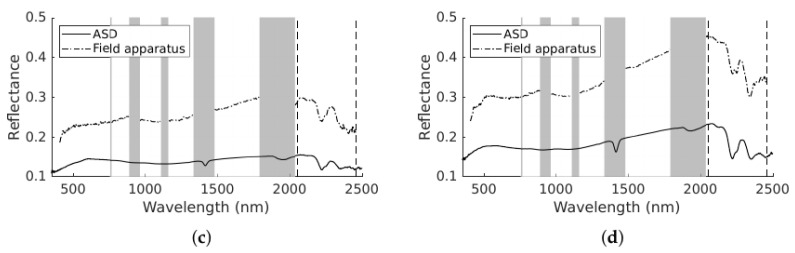
Sample spectra scanned with the Analytical Spectral Devices, Inc. (ASD) TerraSpec FR and the spectral pixel from the scene scanned by the field apparatus corresponding to the approximate location from which the sample was taken. The grey bands represent regions where there is significant atmospheric absorption and data have been removed. The dashed lines at 2050 and 2460 nm show the bounds of the spectral region that was used for classification. (**a**) <0.01 ppm. (**b**) 0.17 ppm. (**c**) 0.71 ppm. (**d**) 1.63 ppm.

Additional ore samples were obtained from this face after the hyperspectral survey. These samples were assayed to determine gold concentration. The approximate location and gold concentration of these samples is also shown in Figure 3. Point spectrometry of the field samples was obtained with an Analytical Spectral Devices, Inc. (ASD) FieldSpec FR in a laboratory on site (see Figure 4 for comparison of the point spectrometer samples and field equipment spectra from the scanned face). These ore samples provide reference data to evaluate the classification of the scanned face.

**Figure 5:**
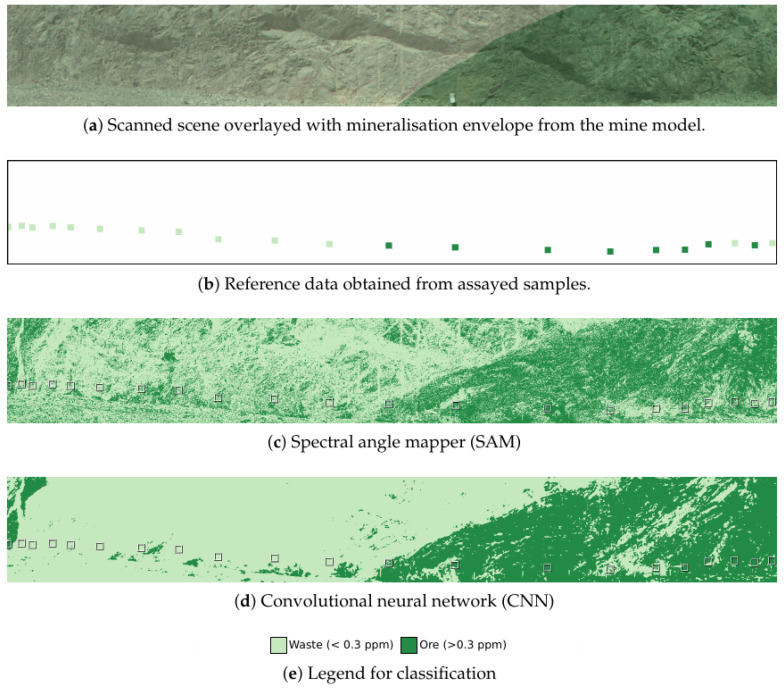
Reference data and results for the binary classification problem with the waste vs. ore boundary at 0.3 ppm. Reference data show the mine mineralisation envelope boundary and the classes of the assayed samples obtained from the face. Results of the classification for both SAM and the CNN are shown. Classification results are coloured by one of the two grade classifications, as shown in the legend, with a bounding box around the regions labelled in the reference model.

The mine provided a geological model which described the mineralization envelope relative to the current state of the mine. The boundary of the mineralization envelope on the scanned face is shown in Figure 5a. This provides another source of reference data to evaluate the classification performance.

### 2.3. Data Preprocessing

Each hyperspectral survey was preceded by a dark current estimation which averaged 200 line scans which were collected with the shutter closed. The camera software removed this measure of background noise from each line scan. The camera calibration files include a non-uniformity matrix that corrects for sensor non-uniformity. This was also corrected in real time by the camera software. Additional background noise in the SWIR camera caused by sensor temperature variation was estimated from the band most affected by atmospheric noise. The across-track brightness gradient in this band was estimated with a first-order polynomial, and along-track with a second order polynomial. This estimate of the background noise variation was subtracted from every spectral band in the SWIR camera. This did not correct illumination variation in the scene due to varying cloud cover or shadowing effects.

The empirical line method [45] was used to convert the image to relative reflectance. For each spectral band, a gain and offset were computed by fitting a first-order polynomial to the at-sensor radiance and the known reflectance of the spectral targets. In the case that the 99% target was saturated, only the 50% target was used, with the assumption that the fit passes through the origin. Data within the atmospheric absorption bands 760–770 nm, 890–965 nm, 1110–1160 nm, 1355–1480 nm, and 1790–2035 nm, were removed. Bands at wavelengths longer than 2460 nm were removed due to decreased illumination and sensor efficiency. No further spectral or spatial smoothing or denoising was applied.

The cameras provided a strong signal in the region of interest, as shown by the signal to noise ratio (SNR) of 195:1 for the field image and 185:1 for the laboratory scanned samples. The SNR of the images was established by dividing the mean by the standard deviation of the reflectance in a spatially uniform part of the scan [11]. This was performed using the 50% reflectance panel, and averaged over the bands used for classification, 2050–2460 nm. This method does not address uncertainty as a result of the background noise correction, nor does it account for illumination variation over the scene, so this does not capture the full uncertainty of the derived reflectance spectra.

### 2.4. Classification

Two classification methods are compared: the spectral angle mapper (SAM), and a convolutional neural network (CNN). The laboratory scans of the ore samples (as shown in Figure 2) were used as reference and training data for both methods, with each pixel of the 2D image labelled with a class determined by the gold concentration of the ore sample. As the number of ore samples are small and not a contiguous sampling of the full range of possible gold concentration, the labels were grouped into classes defined by ranges of gold concentration. For the first problem, binary classification, each input was labelled ore or waste, depending on whether the gold concentration was above or below 0.3 ppm, the threshold used by the geological model. A second classification attempt was performed to test the sensitivity of the classifiers, with the data split into four classes: less than 0.01 ppm, 0.01–0.3 ppm, 0.3–1.0 ppm, and greater than 1.0 ppm.

SAM [46] is a supervised classification method that treats each spectral signature as an n-dimensional vector, and computes the angle between each pixel in a scene and a library of reference spectra:

(1) $$\theta =\cos^{-1}\left(\frac{\sum_{i=1}^{n}t_{i}r_{i}}{{\left(\sum_{i=1}^{n}t_{i}^{2}\right)}^{1/2}{\left(\sum_{i=1}^{n}r_{i}^{2}\right)}^{1/2}}\right),$$

where θ is the spectral angle, *t* is a spectral pixel in the image, *r* is a reference pixel from the library, and *n* is the number of spectral bands. The smaller the value of θ, the more similar the spectra. To classify a pixel, the label of the closest reference spectra is selected. Barton et al. [27] found that classification performance was improved when using samples obtained from the site, rather than from generic spectral mineral libraries. There are a combined 170,000 spectral pixels from the two-dimensional laboratory scans of the 26 ore samples, which is an unwieldly number of reference spectra to compare against to classify a single spectral pixel. As a result, the scans were sub-sampled to generate the library of reference spectra. 50 pixels were randomly selected from each ore sample, and each were labelled with the corresponding gold concentration class and added to the spectral library.

Convolutional neural networks are a series of learned filters which are convolved over the spectral and potentially spatial dimensions of the data to perform classification. Joint spatiospectral models, such as the architecture developed by Ben Hamida et al. [36], operate on a small spatial neighbourhood of each pixel to classify the central pixel. This approach applies 3D convolution filters that gradually reduce the 3D input to a 1D vector. The output of the CNN is a vector containing the output class probability for each class. The class with the highest probability is the chosen label. The six-layer network architecture from [36] was used with a 5 × 5 spatial input patch, where the output class was used to label the central pixel of each patch. This assumes that nearby pixels have similar ore grade and that there is value in preserving local spatial information. The stochastic gradient descent (SGD) solver with an initial learning rate of 0.002 and momentum of 0.9 was used in the learning process. All other common parameters were set as in [36]. The model was implemented and trained in Python with TensorFlow 2.5 [47].

One ore sample in each classification band was set aside for the validation set. The remaining ore samples were used to generate the training set. Each hyperspectral image was decomposed into overlapping 5 × 5 spatial patches (containing the full spectral data for that spatial region) as the input to the network, and the corresponding gold concentration class used as the output label. The CNN was trained with a batch size of 512 over 800 epochs. To avoid overfitting, the solution with the highest validation accuracy was used to classify the field scan.

Both classification methods were performed on the spectral data in the 2050–2460 nm wavelength range, as this band contains the spectral absorption features that were not obscured by atmospheric absorption bands, as seen in Figure 4.

## 3. Results

The ability to discriminate ore from waste was tested by performing a binary classification of the scanned face. All training samples with a gold concentration below 0.3 ppm were labelled as waste, and anything above this as ore. This is the same boundary as the mineralisation envelope used by the geological model. The terrain model of the scanned scene was converted to the mine map coordinates, allowing the mineralisation envelope to be overlaid on the scan, as seen in Figure 5a. Figure 5b shows the reference data constructed from the assayed samples taken from the face. The approximate location of the sample and a window of ten pixels in each direction were labelled as ore or waste by the same 0.3 ppm cut-off. Due to the uncertainty in the exact location and the uniformity of the gold concentration in the local area of each sample, this is better referred to as reference data than as “ground truth”. There are discrepancies between the two sources of reference data. Two samples from the assayed reference data at the right of the scene are classified as waste, despite having been obtained from the mineralised region of the geological model. This likely represents actual variation in gold content not captured by the geological model.

Both SAM and CNN classification results (see Figure 5c,d) have a diagonal ore/waste boundary in the centre of the scanned scene. This is near the boundary that is present in the geological model. The significant difference between the SAM and CNN classified images is the spatial consistency. In the CNN classification, nearby pixels often have the same class. This is the expected outcome of classifying each pixel based on an overlapping patch of pixels, as the patches of adjacent pixels will contain overlapping information. The per-class and overall accuracy relative to the reference assay data are given in Table 1. Both methods classify ore in the waste region and waste in the ore region that do not match the reference data. The CNN outperforms the SAM classification for both classes and overall accuracy, which is expected, given the reduced noise in the classification.

**Figure 6:**
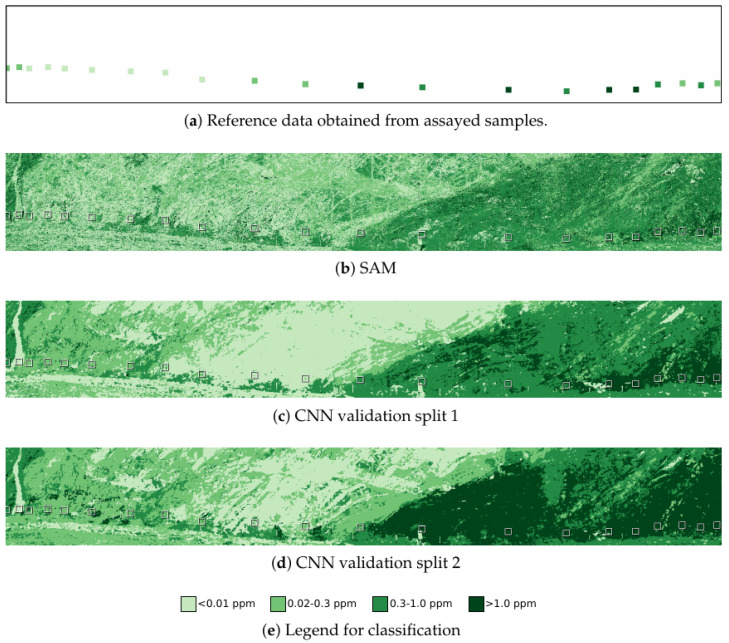
Reference data and results for the four grade classification problem. Reference data show the classes of the assayed samples obtained from the face. Results of the classification for both SAM and the CNN are shown. Classification results are coloured by one of the four grade classifications, as shown in the legend, with a bounding box around the regions labelled in the reference model.

Classification into four ore grade bands was used to test the sensitivity to lower and higher grades of ore. One laboratory sample from each grade band was randomly selected as a validation sample, with two testing/validation splits of the laboratory dataset. In addition to the 0.3 ppm boundary of the mineralisation model, this model further discretised the waste category into above and below 0.01 ppm, the limit of detectable gold in the assay results, and the ore category into above and below 1.0 ppm, as high and low grade ore. These are arbitrary selections which could be varied in response to cut-offs for economically viable excavation, for example. Figure 6 shows the reference data of the assayed samples, now discretised to these four classes, along with the SAM and CNN classification results. Table 2 shows the per-class and overall accuracy relative to the reference data. Accuracy relative to the assayed samples is low, although the feature corresponding to the mineralisation envelope boundary is still visible. Furthermore, the high grade and low grade classifications are sensitive to the split of the laboratory samples into training and validation splits, as seen in Figure 6c,d and in the per-class accuracy. In the second training split, significantly more of the scanned scene is classified as high grade.

**Figure 7:**
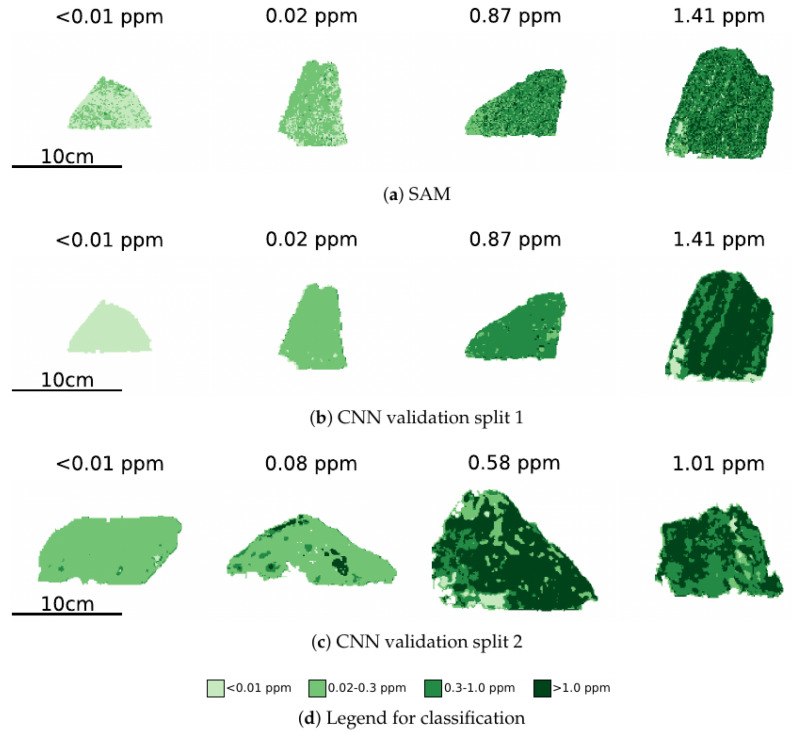
Four grade classification on validation sets. Classification results are coloured by one of the four grade classifications, as shown in the legend.

Similar sensitivity to the training and validation split is seen in the classification performance on the validation datasets (see Figure 7). For the first data split (see Figure 7b), each sample is predominantly classified with the correct label. However, for the second data split (see Figure 7c), both waste classes are largely classified the same, and more of the lower grade ore is classified as high grade ore than the high grade sample. This suggests a large amount of intra-class variability, and is also a limitation of the small number of samples collected prior to fieldwork.

## 4. Discussion

Using hyperspectral imaging to identify ore grade relies on several assumptions: (i) there is a relationship between ore grade and the spectral features of the ore; (ii) these features are detectable by the equipment and have been captured in the data; and (iii) the training data and labels are a representative sample of both the possible variation and the scene being classified.

Relationships between spectral features and gold mineralisation vary from deposit to deposit. Gold itself is not visible at the concentrations present in the samples or the scanned face, so the spectral features of the mineralised rock are being observed. A link between the ore grade and the geological characteristics of the rock is assumed based on the geology of the mine site deposit.

The point spectrometer scans shown in Figure 4 show that the majority of spectral features are in the SWIR region. While the features in the 1400 nm and 1900 nm region are in the atmospheric absorption bands, the features above 2000 nm can be seen in the field spectra, though at lower spectral resolution than the point spectrometry obtained in the laboratory. Above 2400 nm, the features in the field spectra are degraded by noise. This could be addressed with better integration time settings to favour high dynamic range in the >2000 nm region, at the expense of saturation in the more sensitive and better-illuminated bands. Nevertheless, the deployed system under natural solar illumination is able to capture similar features to the point spectrometer under laboratory conditions.

There are two factors to consider in whether the training data provides sufficient coverage of the test space. Firstly, whether the mineralogical content reflects that in the field, and secondly, whether the imaging conditions in the laboratory are sufficiently similar to field conditions. There were 26 discrete samples obtained and scanned for the training set, ranging from lower than detectable gold concentration to 3.05 ppm. Only four samples were over 1 ppm (labelled as high grade for the four grade classification task), while field samples were found to have a gold concentration up to 16 ppm. High-grade ore is under-represented in the training samples.

Comparing the conditions of the laboratory and field scans, the samples in the laboratory were clean, dry samples, scanned under broad-spectrum artificial illumination. In the field, the mine face had areas of moisture, and post-blast excavation areas are likely to be dusty. Shadowing in rocky terrain and varying illumination due to cloud cover were confounding effects that were not corrected. Despite these impacts, the CNN classification algorithm was not obviously sensitive to orientation or shadowing in the field scan. Some misclassification of ore as waste occurred in pixels that are darker relative to their surrounds. It was observed in the field that the mine face was wet in this region. Adding samples to the training set that were washed prior to imaging could make the classifier more robust to the effect of moisture in the field.

The four grade classification problem had some predictive power on the validation dataset, although this was sensitive to the validation data split. The classification of the field scan could not reliably identify the finer split of ore grade. This suggests that while a mineralogical relationship may exist that reflects ore grade, it is not adequately captured in the training dataset. Fire assay has a lower resolution than hyperspectral imaging; while the cameras were able to obtain spectra at the millimetre scale, the assay process returned a single gold concentration for a rock sample. Labelling each spectral pixel in the sample with the same uniform gold concentration may be missing real variation in gold concentration or mineralisation. Mineralisation characteristics, and the relationship between mineralogy and ore grade, may also vary throughout the site, either with depth or location. Assembling a training set that is geolocated would allow separate classifiers to be trained on specific regions in order to detect local variations in mineralisation.

## 5. Conclusions

Both classification algorithms that were tested distinguished ore from waste to the same level as the mine model, without needing to extract spectral features or learn relationships directly from the reflectance spectra itself. This is a novel result in a field application, as prior attempts to discriminate ore and waste in gold samples have largely employed core scans or samples imaged under laboratory conditions. The attempt to discriminate higher or lower ore grades with finer resolution showed low accuracy relative to the reference gold concentration values of samples collected from the scanned mine face. A major limitation of this study is the small number of ore samples available for training data. A larger dataset would better demonstrate the limits of ore grade discrimination with this apparatus and scanning method. The results nevertheless suggest that it is possible to use hyperspectral data to discriminate ore and waste at the point of excavation. While this motivates the need for further investigation, on the evidence presented, the answer to the question that titles this paper is affirmative for gold with a high prospect of extending, *mutatis mutandis*, to other minerals.

## Figures and Tables

**Table 1:** Per-class accuracy and overall accuracy (OA) of classification relative to reference data for the binary classification problem.

Class	SAM	CNN
<0.3 ppm	69.3%	79.2%
>0.3 ppm	45.6%	72.8%
OA	60.1%	76.7%

**Table 2:** Per-class accuracy and overall accuracy (OA) of classification relative to reference data for the four grade classification problem. CNN 1 and CNN 2 refer to the two validation splits.

Class	SAM	CNN 1	CNN 2
<0.01 ppm	50.3%	12.5%	2.4%
0.02–0.3 ppm	25.5%	2.1%	19.1%
0.3–1.0 ppm	47.5%	41.8%	2.3%
>1.0 ppm	21.5%	47.9%	72.7%
OA	37.4%	22.2%	20.6%

## Data Availability

The data generated in this study are openly available in UQ eSpace at https://doi.org/10.48610/42d4d33 (accessed on 24 February 2022).

## Abbreviations

The following abbreviations are used in this manuscript:

CNN	Convolutional neural network
DOF	Degree of freedom
FOV	Field of view
LiDAR	Light detection and ranging
OA	Overall accuracy
RTK GNSS	Real-time kinematic global navigation satellite system
SAM	Spectral angle mapper
SWIR	Short-wavelength infrared
VNIR	Visible-near-infrared
